# Investigation of exotic stable calcium carbides using theory and experiment

**DOI:** 10.1038/ncomms7974

**Published:** 2015-05-11

**Authors:** Yan-Ling Li, Sheng-Nan Wang, Artem R. Oganov, Huiyang Gou, Jesse S. Smith, Timothy A. Strobel

**Affiliations:** 1 Laboratory for Quantum Design of Functional Materials, School of Physics and Electronic Engineering, Jiangsu Normal University, 221116 Xuzhou, China; 2 Department of Geosciences, State University of New York, Stony Brook, New York 11794–2100, USA; 3 Center for Materials by Design, Institute for Advanced Computational Science, State University of New York, Stony Brook, New York 11794-2100, USA; 4 Moscow Institute of Physics and Technology, 9 Institutskiy lane, Dolgoprudny city, Moscow Region 141700, Russia; 5 Northwestern Polytechnical University, Xi'an 710072, China; 6 Geophysical Laboratory, Carnegie Institution of Washington, Washington, District of Columbia 20015, USA; 7 High Pressure Collaborative Access Team, Geophysical Laboratory, Carnegie Institution of Washington, Argonne, Illinois 60439, USA

## Abstract

It is well known that pressure causes profound changes in the properties of atoms and chemical bonding, leading to the formation of many unusual materials. Here we systematically explore all stable calcium carbides at pressures from ambient to 100 GPa using variable-composition evolutionary structure predictions using the USPEX code. We find that Ca_5_C_2_, Ca_2_C, Ca_3_C_2_, CaC, Ca_2_C_3_ and CaC_2_ have stability fields on the phase diagram. Among these, Ca_2_C and Ca_2_C_3_ are successfully synthesized for the first time via high-pressure experiments with excellent structural correspondence to theoretical predictions. Of particular significance is the base-centred monoclinic phase (space group *C*2*/m*) of Ca_2_C, a quasi-two-dimensional metal with layers of negatively charged calcium atoms, and the primitive monoclinic phase (space group *P*2_1_*/c*) of CaC with zigzag C_4_ groups. Interestingly, strong interstitial charge localization is found in the structure of *R*-3*m*-Ca_5_C_2_ with semi-metallic behaviour.

## Abstract (web summary)

Pressure causes profound changes in the properties of atoms and chemical bonding leading to unusual materials. Here, the authors investigate the Ca-C system and find that it becomes increasingly complex and develops a multitude of phases with various compositions and new structures at higher pressures.

## Graphical abstract

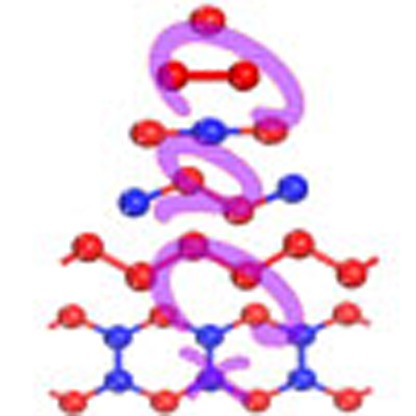

Unexpected chemical reactions can happen under extreme conditions, with emergence of rich phase diagrams and materials possessing intriguing properties. Recently, by combining variable-composition structure prediction methods with first-principles total energy calculations^1^, pressure-composition (*P-x*) phase diagrams were predicted for such binary systems as Mg-O (ref. 2) and Na-Cl (ref. 3). In both cases, the predicted unexpected compounds have been successfully synthesized^3^,^4^. Elemental carbon and calcium both exhibit rich diversity of stable and metastable phases under pressure^5^,^6^,^7^. Compressed calcium shows unique structural and electronic properties and exhibits the highest recorded superconducting critical temperature among pure elements^8^. For carbon, only graphite and diamond are experimentally known as thermodynamically stable solids (graphite is thermodynamically stable at ambient condition and diamond under high pressure), although numerous metastable phases are known. For example, by applying pressure to graphite at low temperatures, a new superhard carbon allotrope was found, and its properties match those of one of the theoretically predicted structures (M-carbon)^9^,^10^,^11^,^12^. For the Ca-C system, the well-known Ca carbides include CaC_2_ and CaC_6_, whose high-pressure behaviours have been studied experimentally^13^,^14^,^15^ and by *ab initio* calculations^16^,^17^,^18^,^19^,^20^. An interesting structural evolution has been uncovered under pressure: carbon atoms polymerize from dumbbells to one-dimensional (1D) chains to ribbons to two-dimensional (2D) graphene sheets in compressed CaC_2_ (ref. 16) and from graphite sheets to a mixed *sp*^2^–*sp*^3^ structure in CaC_6_ (ref. 20). In addition, superconductivity was predicted in metallic high-pressure phases of CaC_2_ with critical temperatures comparable to those observed in CaC_6_ (ref. 16).

Here, using variable-composition structure prediction code USPEX^1^,^9^,^21^, the pressure-composition phase diagram of the Ca-C system was explored in order to fully understand the structural diversity and evolution of the C–C bonding types under high pressure. This resulted in five newly predicted stable stoichiometries (Ca_5_C_2_, Ca_2_C, Ca_3_C_2_, CaC and Ca_2_C_3_) with diverse carbon arrangements: isolated atoms in Ca_2_C, hitherto unknown zigzag tetramers in CaC, and ribbons consisting of five-membered rings in CaC_2_. Two phases (Ca_2_C and Ca_2_C_3_) were confirmed experimentally via *in situ* synchrotron powder X-ray diffraction (PXRD) measurements. Most surprising is that the low-pressure phase (monoclinic *C*2*/m* structure) of Ca_2_C exhibits quasi-2D metallic behaviour and contains negatively charged calcium atoms. In addition, strong interstitial electron localization was found in the newly predicted *R*-3*m* phase of Ca_5_C_2_, just as in compressed elements Li (ref. 22), Na (ref. 23) and Ca (ref. 6), as well as in the compound Mg_3_O_2_ (ref. 2).

## Results

### Convex hull

**Figure 1:**
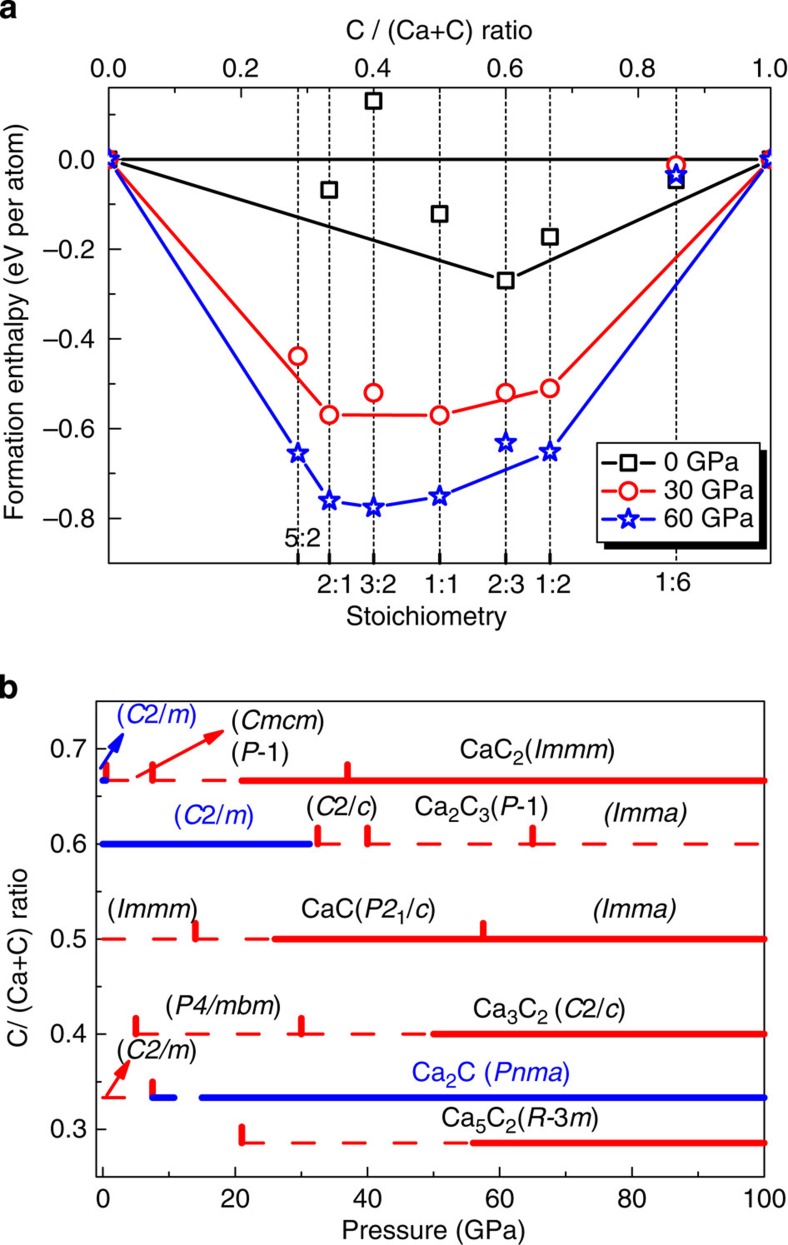
Stability of new calcium carbides. (**a**) Convex hull diagram for the Ca-C system at selected pressures. At a given pressure, the compounds located on the convex hull are thermodynamic stable. (**b**) Pressure-composition phase diagram of the Ca-C system. Thick solid lines represent thermodynamically stable phases and dashed lines represent metastable phases (Red lines represent metal and blue semiconductor).

We have used the *ab initio* evolutionary algorithm USPEX^1^,^9^,^21^, which can simultaneously find stable stoichiometries and the corresponding structures in multicomponent systems, to explore stable Ca-C compounds and their structures. In these calculations, all stoichiometries were allowed (with the constraint that the total number of atoms in the unit cell be below 16 atoms), and calculations were performed at 10, 20, 40, 80 and 100 GPa. The pressure-composition phase diagram of the Ca-C system is given in Fig. 1a, in which the convex hull was obtained from the calculated enthalpies of the most stable structures for each composition at a given pressure. Thermodynamically, the convex hull at a given pressure connects the phases that are stable against decomposition into other binaries or the elements.

### Thermodynamically stable or metastable phases

**Figure 2:**
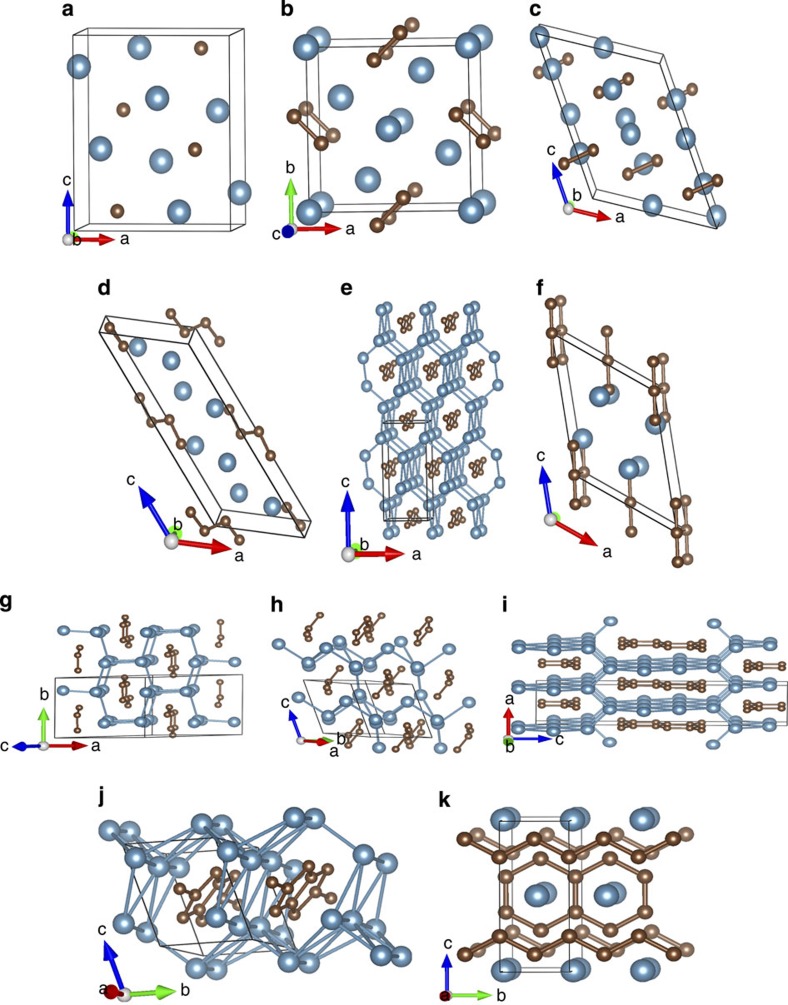
The predicted crystal structures of stable Ca-C compounds. (**a**) Thermodynamically stable *Pnma* structure of Ca_2_C. (**b**) The metastable low pressure *P*4/*mbm* structure of Ca_3_C_2_. (**c**) Thermodynamiclly stable high pressure *C*2/*c* structure of Ca_3_C_2_. (**d**) Thermodynamically stable *P*2_1_/*c* structure of CaC. (**e**) Thermodynamically stable high pressure *Imma* structure of CaC. (**f**) Ca_2_C_3_ crystallizes in *C*2/*m* structure at pressures up to 28 GPa. (**g**) Thermodynamically metastable *C*2/*c* structure of Ca_2_C_3_. (**h**) Thermodynamically metastable *P*-1 structure of Ca_2_C_3_. (**i**) Thermodynamically metastable high pressure *Imma* of Ca_2_C_3_. (**j**) Thermodynamically stable *P*-1 structure of CaC_2_. (**k**) Thermodynamically stable *Immm* structure of CaC_2_. The blue and brown spheres represent calcium and carbon atoms, respectively.

Using variable-composition evolutionary searches, we found that Ca_5_C_2_, Ca_2_C, Ca_3_C_2_, CaC, Ca_2_C_3_ and CaC_2_ have thermodynamic stability fields on the phase diagram: Ca_2_C_3_, stable from 0 to 28 GPa; Ca_5_C_2_, stable above 58 GPa; Ca_2_C, stable above 14 GPa; Ca_3_C_2_, stable from ∼50 GPa; CaC, stable above 26 GPa; and CaC_2_, stable above 21 GPa (see Figs 1b and 2). All crystallographic parameters are listed in Supplementary Table 1. For all the newly predicted structures, calculated phonon dispersion relations confirmed their dynamical stability (see Supplementary Figs 1–5 and Supplementary Note 1). Surprisingly, our theoretical calculations show that the known phases of CaC_2_ and CaC_6_ are thermodynamically metastable at normal conditions (see Fig. 1a); CaC_2_ is thermodynamically stable only above 21 GPa, and CaC_6_ does not have a thermodynamic stability field (BaC_6_ is thermodynamically stable in the Ba-C system at 1 atm (ref. 24)). We also explored metastable phases of Ca_2_C and CaC at lower pressure. The most stable low-pressure phase obtained for Ca_2_C has *C*2*/m* symmetry and that of CaC has *Immm* symmetry. The dynamical stability of these two thermodynamically metastable phases was confirmed via phonon calculations (Supplementary Figs 6 and 7).

**Figure 3:**
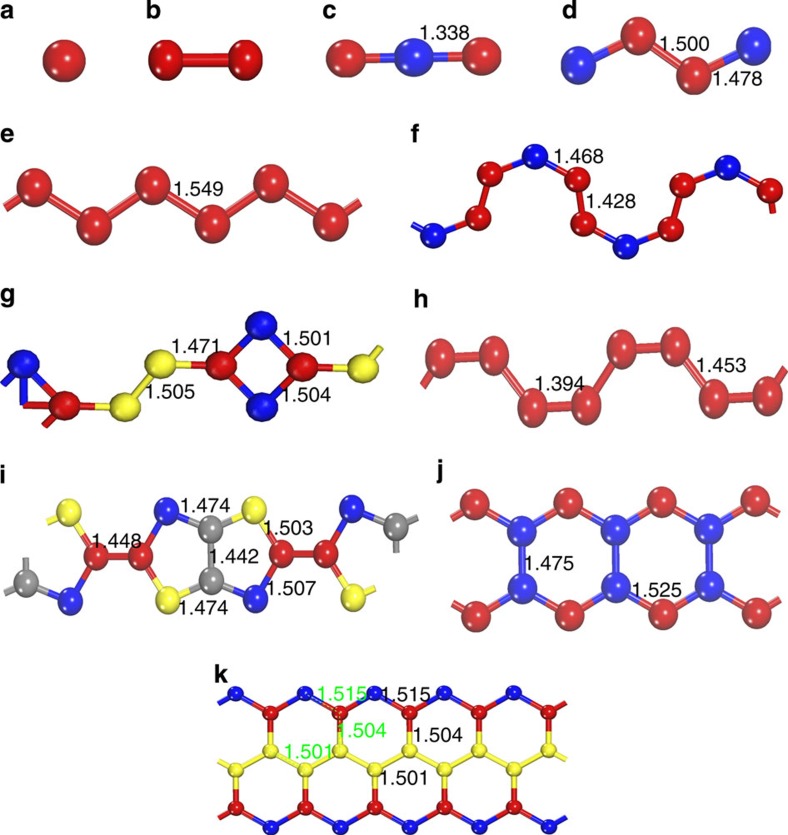
Carbon patterns in the Ca-C system. (**a**) The isolated carbon anions in the *Pnma* structure of Ca_2_C. (**b**) Carbon dimers observed in the *C*2/*m* structure of Ca_2_C, *P*4/*mbm* and *C*2/*c* structures of Ca_3_C_2_, *Immm* structure of CaC, and *C*2/*m* or *C*2/*c* structures of CaC_2_. (**c**) The carbon trimer occurs in the *C*2/*m* structure of Ca_2_C_3_ at zero pressure. (**d**) Zigzag C_4_ groups observed in *P*2_1_/*c* structure of CaC at 20 GPa. (**e**) Zigzag carbon chains in the *Imma* structure of CaC at 58 GPa. (**f**) Carbon chains with two types of carbon-carbon bondings in *C*2/*c* structure of Ca_2_C_3_ at 34.5 GPa. (**g**) Carbon chains with four types of carbon-carbon bongdings in *P*-1 structure of Ca_2_C_3_ at 40 GPa. (**h**) Armchair carbon chains in the *Cmcm* structure of CaC_2_ at 4 GPa. (**i**) Carbon stripes in the *P*-1 structure of CaC_2_ at 20 GPa. (**j**) Carbon ribbons in the *Immm* structure of CaC_2_ at 10 GPa. (**k**) Carbon ribbons in the *Imma* structure of Ca_2_C_3_ at 65 GPa. Bond lengths (in Å) are given. The inequivalent C1, C2, C3, and C4 (occupying different Wyckoff positions in the unit cell, see Supplementary Table 1) are shown by red, blue, yellow, and grey spheres, respectively.

**Figure 4:**
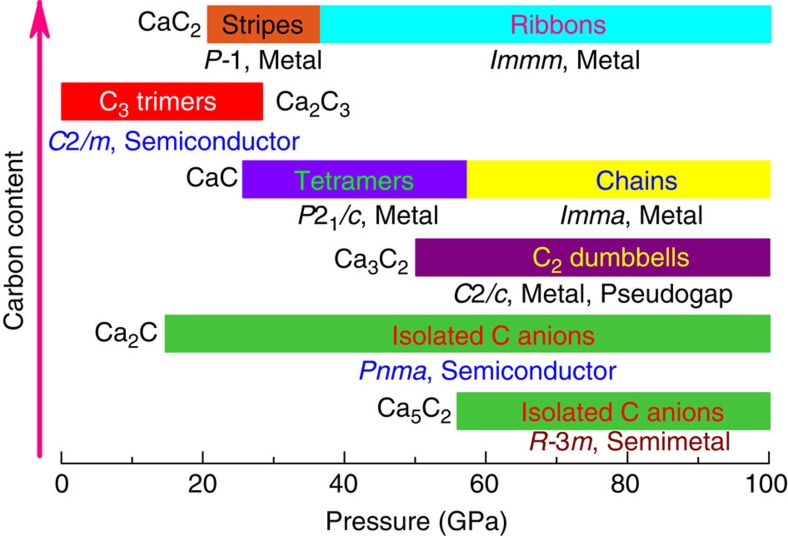
Carbon arrangement with increasing carbon content. Only thermodynamically stable phases are shown (For metastable phases, see Supplementary Table 2). Additionally, the conducting properties are shown.

In order to analyse these predicted structures, we recall that the C–C bond length depends on the bond order, and at 1 atm these lengths are 1.20 Å for the triple C–C bond, 1.33 Å for double bond and 1.54 Å for single C–C bond. The carbon patterns predicted for calcium carbides, on the basis of calculations presented in this work, are plotted in Fig. 3. The carbon patterns together with their pressure range of existence in calcium carbides are summarized in Fig. 4 and in Supplementary Table 2. Combining this knowledge with the results of Bader analysis (see Supplementary Table 1), we unravel very diverse chemistry. From the results of the Bader analysis, one can clearly see the correlation between the charge and volume: negatively charged calcium atoms occupy significantly greater volume. In addition, we observe the decrease in C–C bond order from triple to double to single bonds as pressure increases. Note, however, that at pressures up to 100 GPa, the carbon-richest stable compound is CaC_2_. Below we consider the predicted phases in order of increasing carbon content.

### Ca_5_C_2_

**Figure 5:**
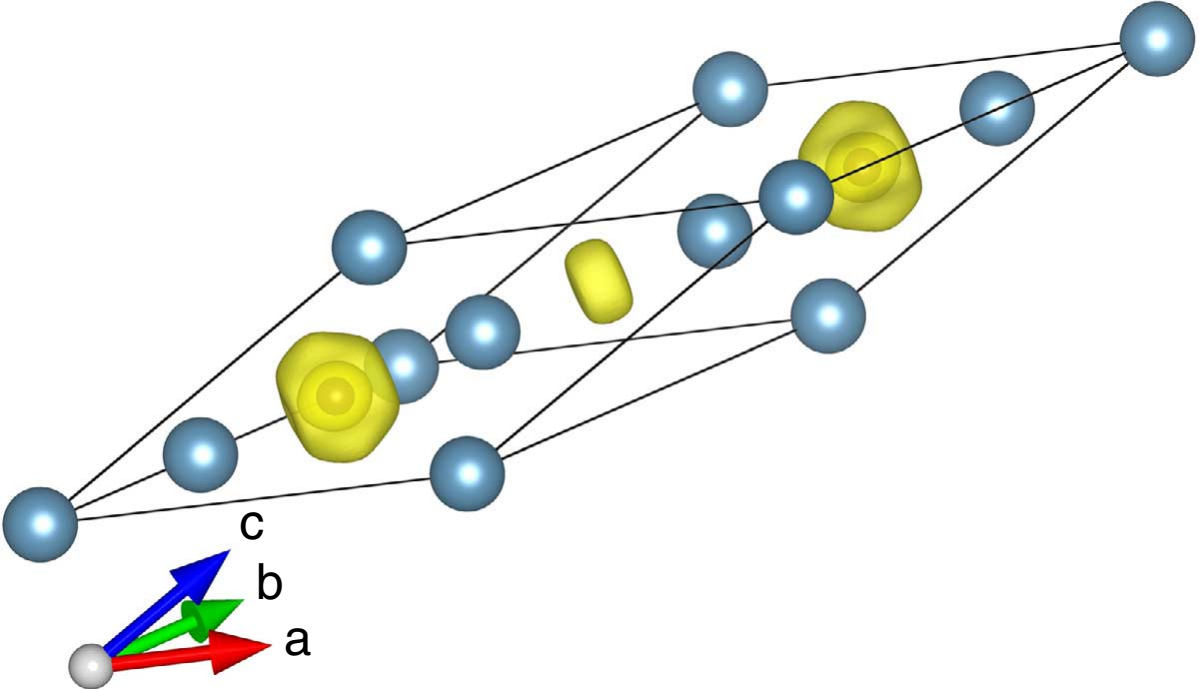
Electron localization function (ELF) of *R*-3 *m*-Ca_5_C_2_ at 60 GPa. The isosurface ELF=0.75 is shown. The observed interstitial electron charge accumulation shows that Ca_5_C_2_ with *R*-3 *m* symmetry is an electride.

The stable structure of Ca_5_C_2_ has a *R*-3*m* symmetry. It is a semi-metal (see Supplementary Fig. 8) and is thermodynamically stable at pressures ranging from 58 GPa to at least 100 GPa (see Fig. 4). This phase has novel structural features: it can be described as consisting of alternating CaC_2_ layers (where Ca is octahedrally coordinated by C atoms) and layers with composition Ca_4_. The electron localization function distribution in Ca_5_C_2_ shows strong charge transfer from Ca to C. Non-nuclear charge density maxima are located in the Ca_4_ layer as plotted in Fig. 5 (electron localization function=0.75 at 60 GPa). Bader charges are +1.039 for Ca1, +0.823 for Ca2, +0.973 for Ca3 and −0.459 for the interstitial electron density maximum.

### Ca_2_C

**Figure 6:**
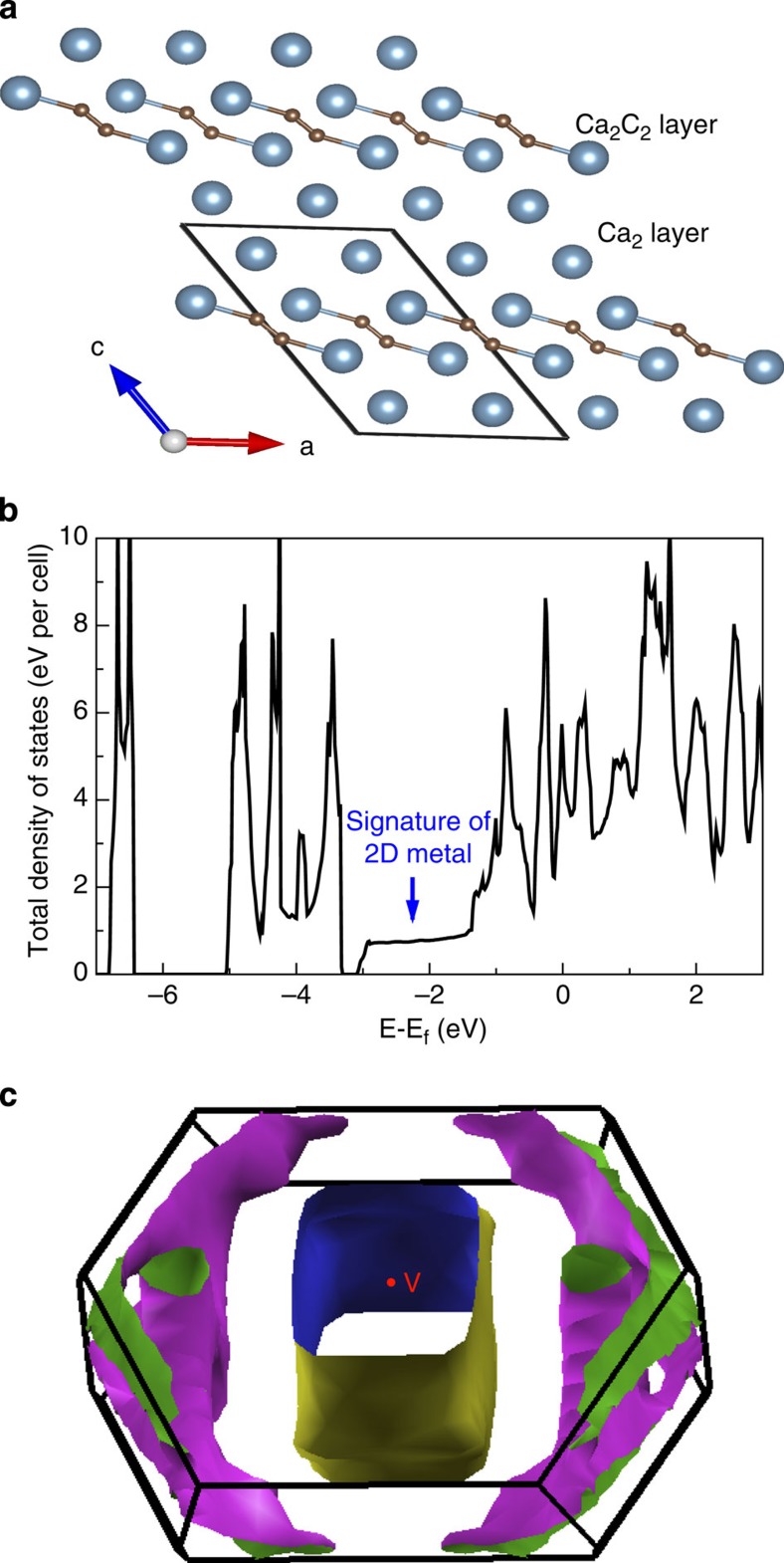
Structural and electronic property of *C*2*/m* phase of Ca_2_C at 3 GPa. (**a**) The top view of *C*2*/m* structure along the y axis shows a clearly layered structure. (**b**)Total electronic density of states (DOS). (**c**) Fermi surface of the *C*2/*m* structure. The indication of a quasi-two-dimensional metal in total DOS is confirmed by the hollow square prismatic cylinder-like Fermi surface.

Known alkali earth methanides include well-know Be_2_C (*Fm*-3*m*, Z=4) and Mg_2_C (antifluorite) recently synthesized by Kurakevych *et al*.^25^ However, no theoretical or experimental information has been reported on the methanide Ca_2_C (ref. 26). According to our calculations, Ca_2_C is thermodynamically stable above 15 GPa (space group *Pnma* (Z=4)). For *Pnma*-Ca_2_C we observe the largest negative charge of carbon atoms among all these phases: −2.321. In this semiconducting phase with band gap of 0.64 eV at 14 GPa (see Supplementary Fig. 9), C atoms are isolated and one can represent this compound as a carbide with an idealized charge transfer scheme (Ca^2+^)_2_C^4−^ adhering to the Zintl concept. Metallic metastable *C*2*/m*-Ca_2_C has a unique structure, consisting of alternating layers of stoichiometry Ca_2_(C_2_) and Ca_2_ (two kinds of calcium atoms play distinctly different roles, see Fig. 6 a), and these layers have net charges of +0.582 and −0.582, respectively (see Supplementary Table 1). What is unusual is that the Ca layer is negatively charged, that is, it is a reservoir of electrons. To further analyse this phenomenon, it is instructive to look first at the Ca_2_(C_2_) layer. This C_2_ group can be represented as having a triple C–C bond and its ideal charge is −2 (Bader charge is −1.892), and if each Ca had the ideal charge of +2, the total charge of the Ca_2_(C_2_) layer would be +2, and two electrons would be transferred to the Ca_2_ layer (see Supplementary Table 1). In reality, the C–C bond here has a somewhat lower order (C–C distance is 1.28 Å at 5 GPa) and therefore takes more electrons from Ca atoms, leaving less for the Ca_2_ layer, but not changing the picture qualitatively. To our knowledge, this is the first example of negatively charged metal atoms in a compound with more electronegative atoms. Note the enormous difference of Bader volumes of the positively and negatively charged Ca atoms (16.570 versus 41.901 Å^3^). One can expect that the electrons in the Ca layer are very loosely bound, and the work function of this compound can be expected to be extremely low. The density of states of the metastable *C*2*/m* phase of Ca_2_C reveals a remarkable step-like feature near the bottom of the valence band, followed by a nearly constant density of states (see Fig. 6b), presenting an example of a quasi-2D electronic structure as observed in Li–Be alloys^27^. The calculated Fermi surface of *C*2*/m*-Ca_2_C at 3 GPa has a hollow square cylinder-like Fermi shape along the Γ-V direction (that is, reciprocal lattice basis vector ***b***_3_ direction) in the Brillouin zone (BZ), signalling quasi-2D electronic properties (see Fig. 6c).

### Ca_3_C_2_

For Ca_3_C_2_, no thermodynamically stable phase exists below 50 GPa. A metastable *P*4*/mbm* (Z=2) phase, favoured in the pressure range from 5 to 30 GPa, transforms into a *C*2*/c* (Z=4) structure at 30 GPa, which is thermodynamically stable above 50 GPa. The structure of *P*4*/mbm*-Ca_3_C_2_ contains doubly bonded C_2_ groups (C–C distance 1.39 Å at 20 GPa), with an ideal charge −4, that is, accepting four electrons from calcium atoms, leaving two electrons per formula to form Ca–Ca bonds in this metallic compound. Metallic *C*2*/c*-Ca_3_C_2_ with a pseudogap at the Fermi level (see Supplementary Fig. 10) has singly bonded C_2_ groups (C–C bond length 1.51 Å at 38.7 GPa), which have ideal charge −6, exactly balanced by three Ca atoms in the formula.

### CaC

Metallic CaC has two thermodynamically stable phases below 100 GPa. At 14 GPa, the metastable orthorhombic *Immm* structure transforms into a monoclinic *P*2_1_/*c* structure (stable thermodynamically above 26 GPa, favoured over a wide pressure range of 14–57.5 GPa), followed by a thermodynamically stable *Imma* structure. *P*2_1_*/c*-CaC is very interesting because its structural formula Ca_4_(C_4_) contains a unique and hitherto unknown zigzag C_4_ group, with C–C distances between 1.48 and 1.50 Å at 14 GPa, indicating bond orders between 1 and 2 and ideal charges of about −2.5 for the end C atoms (Bader charge −1.447) and about −1 for the central C atoms (Bader charge −0.905). *Imma*-CaC has infinite zigzag chains of C atoms (C–C bond length of 1.55 Å at 57.5 GPa, indicating a weakened single bond) in the *y* axis direction. The structural formula of metastable *Immm*-CaC is Ca_2_(C_2_), and with a doubly bonded C_2_ group (C–C distance 1.33 Å at 7.1 GPa) that has an ideal charge of −4 (Bader charge –2.340), it exactly balances the ideal charge of two Ca atoms. All three phases of CaC beautifully conform to the trend of increasing polymerization of the C sublattice with increasing pressure.

### Ca_2_C_3_

The structure of Mg_2_C_3_ (space group *Pnnm*, *Z*=2), the only known alkaline-earth metal allylenide with C_3_^4−^ anions^26^, was considered when searching for stable phases of Ca_2_C_3_. Total energy calculations exclude the possibility of the ambient-pressure Mg_2_C_3_-type structure. The semiconducting *C*2*/m* structure (band gap of 1.06 eV at 10 GPa, see Supplementary Fig. 11) is instead the most stable one below 34.5 GPa (thermodynamically stable from 0 to 28 GPa), followed by metastable *C*2*/c* structure. By fitting energy versus volume data to the third-order Birch–Murnaghan equation of state^28^, the calculated bulk modulus *B*_0_ of *C*2*/m*-Ca_2_C_3_ is ∼89 GPa, which is higher than that of CaC_2_ (50 GPa). At ∼40 GPa, the metallic *C*2*/c* structure transforms into a metastable *P*-1 structure (metal), which dominates the pressure range between 40 and 65 GPa. At higher pressures, a metallic metastable *Imma* structure is stable and contains zigzag carbon chains (Figs 3 and 4). We searched at much higher pressures for 3D-polymeric carbon frameworks in Ca_2_C_3_, but found none at pressures up to at least 300 GPa. For comparison, in CaC_2_ we have found that graphene sheets predicted in the high-pressure phase can be stable up to at least 1 TPa (ref. 16).

For Ca_2_C_3_, the carbon arrangement changes from isolated C_3_ to carbon chains to ribbons (Fig. 3). The structure of *C*2*/m*-Ca_2_C_3_ can be described as Ca_2_ layers linked together by nearly linear symmetric C_3_ groups with double C–C bonds (C–C distances 1.32 Å at 18.1 GPa). With this configuration, the total charge of the C_3_ group should be −4 (Bader charge −2.692), exactly compensating the charge of two Ca atoms in the formula (see Supplementary Table 1). Central carbon atoms in the C_3_ group in this valence scheme should be neutral, and yet turn out to have a large negative Bader charge of −0.738, whereas the end atoms, whose idealized charge is −2, develop a lower Bader charge (−0.977). This discrepancy is explained by the effects of Ca atoms, which form significant bonds with the central carbon atom in the C_3_ group and transfer some electronic charge to them. Most recently, some of us reported the prediction and synthesis of β-Mg_2_C_3_ (ref. 29), which is isostructural with our *C*2*/m*-Ca_2_C_3_ reported here. The structure of *C*2*/c*-Ca_2_C_3_ (C–C distances 1.43–1.47 Å at 34.5 GPa) has an idealized charge transfer scheme Ca_2_^4+^C_3_^4−^. In this metallic phase, C atoms are polymerized into infinite chains with nearly closed six-member rings running through channels of Ca host lattice. *P*-1-Ca_2_C_3_ features a complicated extended 1D ribbon of carbon atoms with nearly single C–C bonds (lengths 1.47–1.50 Å at 40 GPa).

*Imma*-Ca_2_C_3_ has a very interesting structure with extended 1D ribbons of carbon atoms cut from the graphene layer. Bond lengths in this ribbon are 1.50–1.52 Å at 70 GPa, slightly longer than in graphene and indicating predominantly single bonds. Electronic structure calculations show that both *P*-1 and *Imma* phases of Ca_2_C_3_ are metals. On the basis of Allen and Dynes modified equation^30^, we have checked for superconductivity in these phases at 34 and 65 GPa, respectively, and found none.

### CaC_2_

CaC_2_ is thermodynamically stable above 21 GPa (see Fig. 1a). The lower-pressure phases *C*2*/m* and *Cmcm* reported previously^16^ are metastable, which could be unravelled by calculating enthalpy of formation (Δ*H*_f_) at lower pressure. Considering that graphite is the ground state of carbon at zero pressure, we performed additional calculations where the van der Waals interaction is accounted for by using the optPBE-van der Waals functional^31^. At zero pressure, the calculated Δ*H*_f_ (−0.17 eV per atom) of CaC_2_ is close to the experimental standard Δ*H*_f_ (−0.21 eV per atom at 298 K and 1 atm (ref. 32)) but higher than that (−0.27 eV) of Ca_2_C_3_, confirming the thermodynamic metastability of CaC_2_ under ambient conditions (see Fig. 1a). It is very unexpected, but the above numbers fully confirm this conclusion, that the well-known and industrially produced compound CaC_2_ is metastable under ambient conditions, while the so far never seen compound Ca_2_C_3_ is actually stable. This could be either due to kinetics, or due to high-temperature conditions of synthesis. In addition to our previous result^16^, we found a new phase with the *P*-1 symmetry, which contains infinite carbon chains with five-membered rings (C–C distance is between 1.442 and 1.507 Å at 20 GPa (see Fig. 3i), signalling single or double bonds), and is the lowest enthalpy structure over a wide pressure range from 7.5 to 37 GPa (thermodynamically stable from 21 to 37 GPa, see Fig. 4 and Supplementary Fig. 12). With further application of pressure, metallic *P*-1-CaC_2_ transforms into metallic *Immm*-CaC_2_ (ref. 16), in which carbon atoms are polymerized to form quasi-1D ribbons (see Figs 2, 3).

### Experiments

In order to confirm theoretical structure predictions, we performed synthesis under high-pressure/high-temperature conditions. Diamond anvil cells were loaded with both calcium- and carbon-rich Ca+C mixtures, compressed to pressures up to 25 GPa, heated to temperatures up to ∼2,000 K and probed *in situ* using synchrotron PXRD. Under these pressure conditions, the formation of *Immm*-CaC_2_, *C*2*/m*-Ca_2_C_3_ and *Pnma*-Ca_2_C may be expected on the basis of thermodynamic stabilities, as these phases are the only stable ones that appear on the convex hull up to 25 GPa (see Fig. 1); indeed, two of these three structures were observed experimentally.

**Figure 7:**
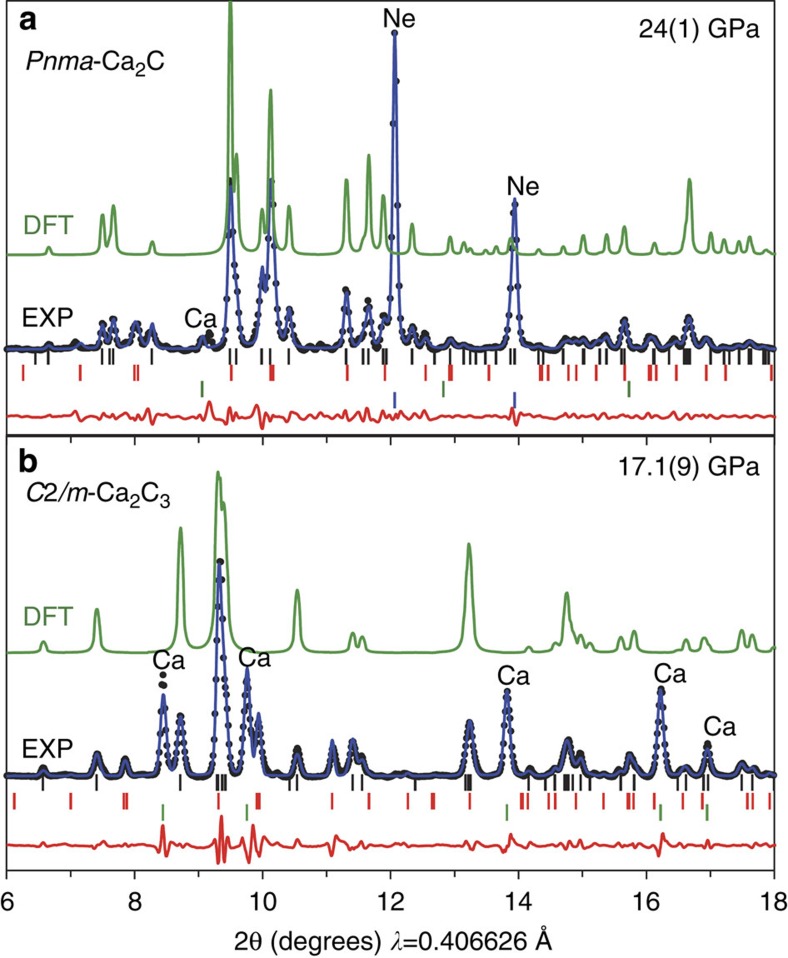
PXRD patterns for experimentally observed Ca-C phases. (**a**) *Pnma*-Ca_2_C synthesized at 24 GPa (*wR*_*p*_=1.5%, *R*_*p*_=0.9%). (**b**) *C*2*/m*-Ca_2_C_3_ synthesized at 17 GPa (*wR*_*p*_=2.0%, *R*_*p*_=0.9%). Experimental data (points) are compared with full-profile refinements using the Le Bail method (blue lines), with differences (red lines). Simulated powder patterns using atomic positions derived from DFT-optimized structures are shown for intensity comparison (green lines). Positions of reflections of Ca_2_C (**a**) or Ca_2_C_3_ (**b**), Ca, Ne and a third carbide phase (see Supplementary Note 2) are indicated by black, green, blue and red tick marks, respectively.

**Figure 8:**
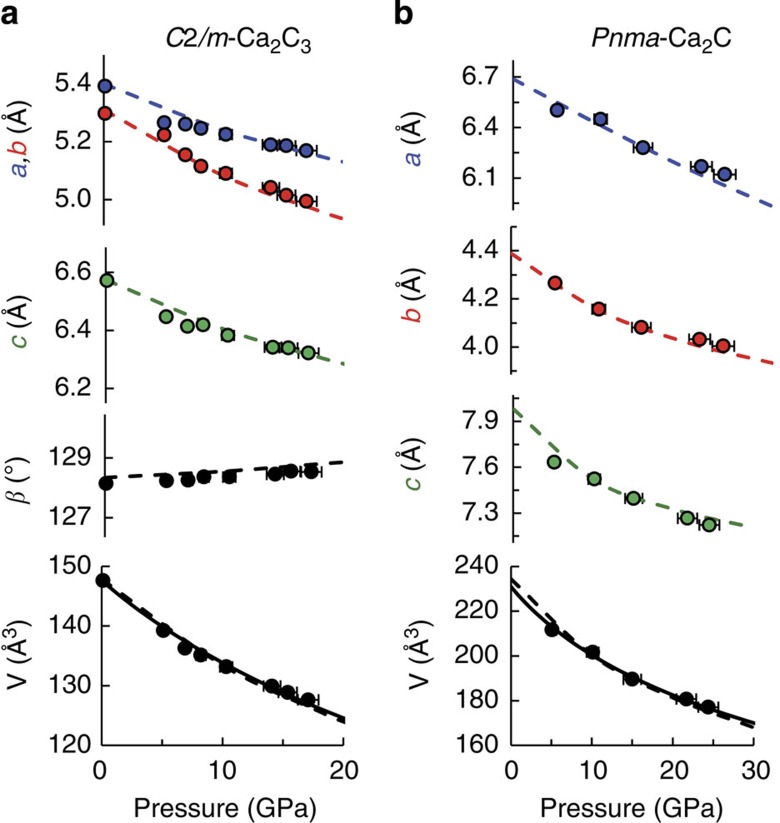
Lattice parameters and unit cell volumes for Ca-C phases. Experimental parameters (points) are compared with DFT predictions (dashed lines) for both *C*2*/m*-Ca_2_C_3_ (**a**) and *Pnma*-Ca_2_C (**b**). Experimentally-derived equations of state for both phases are shown as solid lines in the lower panels.

When samples were compressed above ∼10 GPa and heated to ∼2,000 K, mixtures of elemental glassy carbon and face-centred cubic (*fcc*) Ca transformed into a new low-symmetry phase. After comparison with density functional theory (DFT) structure predictions, PXRD reflections originating from this phase were readily indexed to the monoclinic *C*2*/m*-Ca_2_C_3_ structure with excellent agreement between experiment and theory (see Supplementary Table 3). Figure 7 shows experimental PXRD data obtained at 17 GPa with *a*=5.169(4) Å, *b*=4.994(3) Å, *c*=6.322(3) Å and *β*=128.53(3)°, which compares with *a*=5.151 Å, *b*=4.962 Å, *c*=6.306 Å and *β*=128.81° for the theoretical structure relaxed at 18 GPa. This sample was decompressed in steps of ∼2 GPa to obtain lattice parameters as a function of pressure (see Fig. 8). Theoretical lattice parameters show an average absolute deviation of 0.3% from experiment over the entire pressure range, and the *C*2*/m*-Ca_2_C_3_ phase was recoverable to ambient pressure, but was found to be air/moisture-sensitive. The experimental *P–V* data were fit to a second-order Birch–Murnaghan equation of state with *B*_0_=84(2) GPa, in good agreement with theoretical predictions (89 GPa).

At pressures above ∼22 GPa, a second carbide phase (*Pnma*-Ca_2_C) was synthesized upon laser heating. This same phase was reproducibly formed both from elemental Ca+C mixtures and from samples containing *C*2*/m*-Ca_2_C_3_, indicating disproportionation of Ca_2_C_3_ into a more stable carbide phase when pressure is raised above ∼22 GPa, i.e. above its predicted stability field. Figure 7 shows experimental PXRD data at 24 GPa with *a*=6.122(1) Å, *b*=4.004(1) Å, *c*=7.223(1) Å, which compares with *a*=6.044 Å, *b*=3.977 Å, *c*=7.265 Å for DFT calculations at the same pressure (see Supplementary Table 4). Calculated lattice parameters show an average absolute deviation of 0.5% from experimental values between 25 and 5 GPa (see Fig. 8), which was the lowest pressure obtained due to failure of a diamond anvil. Fitting the P–V data to a second-order Birch–Murnaghan equation of state yields *B*_0_=53(4) GPa.

## Discussion

We find that the carbon sublattice within all predicted carbide phases has close correlation with the Ca:C ratio (see Fig. 2). With increasing carbon content, isolated carbon atoms are polymerized, in turn, into C_2_ dumbbells, C_3_ and C_4_ groups, chains, ribbons and graphene sheets (see Fig. 4 and Supplementary Table 2). The polymeric carbon structures reveal an expected trend when comparing with the structural chemistry of the heavier congeners of group IV elements in Zintl phases (alkali or alkaline-earth silicides, germanides and stannides)^17^,^33^. Yet in spite of certain similarities to silicides, calcium carbides differ from them because of distinct bonding features. Combining present analysis and our previous results^16^,^17^,^20^, one can conclude that for the Ca-C system, one can cover *sp* to *sp*^2^ to *sp*^2^+*sp*^3^ to *sp*^3^ hybridizations of carbon as pressure increases. This pressure-induced structural evolution of carbon was also found in other alkali metal or alkaline-earth metal carbides^17^,^20^,^24^. Together with our previous results for CaC_2_ (ref. 16) and CaC_6_ (ref. 20), it is clear that a 3D network of carbon in CaC_*x*_ can be formed when *x* is greater than 2 (from sheets to 3D frameworks to Ca-C phase separation with slabs of diamond at high C content), consistent also with the behaviour of the metastable CaC_4_ compound found in our structural searches. On the other hand, the structural features of carbon-rich compounds^20^ can be extended to alkali-metal or alkaline-earth metal congeners of the group-IV elements, which allows one to fabricate a variety of the 3D framework structures of the group-IV elements by removing metal sublattices. The unexpected mechanical^20^,^34^ or electronic characteristics^35^ uncovered in these 3D framework structures pave the way to novel materials.

In summary, we have produced the first complete pressure-composition phase diagram for CaC_*x*_ compounds at pressures up to 100 GPa and demonstrated the experimental synthesis of two previously unknown compounds (Ca_2_C_3_ and Ca_2_C), validating part of our predicted phase diagram. Contrary to normal ionic compounds, there is no ‘dominant' compound stable in this whole pressure range. The well-known CaC_2_ and CaC_6_ were found to be metastable at normal conditions; CaC_2_ is stable only above 21 GPa, and CaC_6_ is never thermodynamically stable, while hitherto unreported Ca_2_C_3_ is actually thermodynamically stable at ambient pressure. Bader analysis unravels very diverse chemistry: the decrease in C–C bond order from triple to double to single bonds at increasing pressure; a negatively charged metal layer in calcium-rich Ca_2_C compound; a hitherto unknown bent linear C_4_ group in the *P*2_1_/*c* phase of CaC; C ribbons being present in carbon-rich compounds. The *C*2/*m*-Ca_2_C phase provides a fresh and very attractive example of a 2D metal, presenting the only known example of a compound where a metal atom (Ca) develops a negative Bader charge in presence of a more electronegative atom (C). Such unusual compounds are likely to find potential applications if synthesized in sufficiently large quantities. While powerful computational methods, such as USPEX, are capable of reliably predicting these exotic compounds, simple theoretical models capable of anticipating them are yet to be developed.

## Methods

### Structure search and theoretical calculations

Searches for stable structures of the Ca-C system under compression were carried out using the evolutionary algorithm USPEX in combination with the VASP code^36^ on the basis of DFT within the generalized gradient approximation with the exchange-correlation functional of Perdew, Burke and Ernzerhof^37^, employing the projector-augmented wave^38^ method [He] and [Ne] cores for C and Ca atoms, respectively. For carbon, a ‘hard' PAW potential was used in search for stable structures. For the crystal structure searches, we used a plane-wave basis set cutoff of 700 eV and performed the BZ integrations using uniform Gamma-centred k-point meshes. The most interesting structures were further relaxed at a higher level of accuracy with a basis set cutoff of 1,000 eV and a k-point grid of spacing 2*π* × 0.018 Å^−1^. Iterative relaxation of atomic positions was stopped when all forces were smaller than 0.001 eV Å^−1^. For compounds predicted via variable-composition searches, we re-searched their stable structures using fixed-composition calculations, with two, three and four formula units per unit cell. For Ca_2_C_3_, some evolutionary calculations were also performed under the pressure of 30, 50, 80, 120, 160, 240 and 300 GPa with two or four chemical formula units per unit cell so as to discern the possibility of 3D network carbon.

The enthalpy of formation per atom of Ca_*n*_C_*m*_ is defined as Δ*H*_*f*_(Ca_*n*_C_*m*_)=[*H*(Ca_*n*_C_*m*_)-*nH*(Ca)-*mH*(C)]/(*n*+*m*), where all enthalpies *H* are given at the same pressure and zero temperature. At a given pressure, the calcium carbides located on the convex hull are thermodynamically stable against decomposition to any other binaries or the elements, while the compounds above the convex hull are meta-stable.

The Bader analysis was performed for exploring chemical bonding and local electrons^39^. To get a converged charge density, the plane wave kinetic energy cutoff of 1,000 eV and Monkhorst-Pack **k**-point meshes with the reciprocal space resolution of 2*π* × 0.02 Å^−1^ were used for all the structures. A series of FFT grids to accurately reproduce the correct total core charge were tested by increasing parameters NG(X,Y,Z)F to 1.5, 2 and 2.5 times the default one.

The lattice dynamics and superconducting properties of Ca_2_C_3_ were calculated by the Quantum ESPRESSO package^40^ using Vanderbilt-type ultrasoft pseudopotentials with cutoff energies of 50 and 500 Ry for the wave functions and the charge density, respectively. The electronic BZ integration in the phonon calculation was based on a 12 × 12 × 12 of Monkhorst-Pack k-point meshes. The dynamical matrix was computed on the basis of a 6 × 6 × 6 mesh of phonon wave vectors. The electron–phonon coupling was convergent with a finer grid of 48 × 48 × 48 **k** points and a Gaussian smearing of 0.01 Ry. For other compounds, phonon calculations were performed using the Phonopy code^41^. The Fermi surface of *C*2*/m*-Ca_2_C at 3 GPa was calculated using Quantum Espresso and 16 × 16 × 8 of Monkhorst-Pack k-point mesh.

### Experiment

Reagents for experimental studies consisted of commercial calcium metal (Sigma-Aldrich, dendritic pieces, 99.99%) and glassy carbon (Sigma-Aldrich, 2–12 μm, 99.95%). The carbon powder was degasified for 12 h at ∼200 °C in a vacuum oven and then sealed under Ar. Diamond anvil cell syntheses (up to 25 GPa and 2,000 K with *in situ* PXRD) were performed at High Pressure Collaborative Access Team (HPCAT), sector 16, of the Advanced photon Source. A small amount of carbon powder and Ca metal shavings were pressed in thin layers within a rhenium gasket in a diamond anvil cell equipped with 400-μm culet diamonds inside of an inert Ar glovebox (O_2_<1 p.p.m.; H_2_O<1 p.p.m.). It was not possible to control the precise Ca:C ratio; however, compositions were estimated to range between 0.333≤Ca:C≤3, on the basis of the volume of material loaded into the diamond cell. Samples were sealed inside the glovebox without pressure medium or loaded with Ne to improve thermal isolation from diamonds and quasi-hydrostatic conditions for subsequent diffraction measurements. Pressure was estimated using the Ne equation of state^42^ and/or with a ruby gauge^43^. To generate high temperatures, samples were heated on both sides using infrared fibre lasers. In some cases two or three heating cycles were performed, with X-ray diffraction patterns collected before, during and after laser heating. During laser heating, temperatures from each side of the sample were estimated by collecting emitted thermal radiation, correcting for the optical system response and fitting the spectral data to Planck's equation. Angle-dispersive PXRD patterns were collected using a mar345 detector (marXperts GmbH, Norderstedt, Germany) image plate calibrated with a CeO_2_ standard. Patterns were integrated using FIT2D (ref. 44) and phase recognition and indexing were performed using PowderCell and CheckCell programmes^45^. While observed PXRD intensities were in good agreement with DFT-derived structural models, observed powder statistics were not suitable for Rietveld refinements. Full profile refinements were performed using the Le Bail intensity extraction method, as implemented in GSAS^46^ with EXPGUI^47^ (see Supplementary Figs 13–15 and Supplementary Note 2 for additional details).

## Author contributions

Y.-L.L. and A.R.O. designed the research. T.A.S. designed the experiments. Y.-L.L., A.R.O. and S.-N.W. performed calculations. H.G., J.S.S. and T.A.S. performed the experimental studies. All authors analysed the data and contributed to write the paper.

## Additional information

**How to cite this article**: Li, Y. L. *et al*. Investigation of exotic stable calcium carbides using theory and experiment. *Nat. Commun*. 6:6974 doi: 10.1038/ncomms7974 (2015).

## Supplementary Material

Supplementary Information Supplementary Figures 1-15, Supplementary Tables 1-4, Supplementary Notes 1-2 and Supplementary References
