# Supplementary material for: Investigation of exotic stable calcium carbides using theory and experiment
Source: Nat Commun. 2015 May 11;6:6974. doi: 10.1038/ncomms7974 (PMC4432597; doi:10.1038/ncomms7974)
Supplement: Supplementary Information — Supplementary Figures 1-15, Supplementary Tables 1-4, Supplementary Notes 1-2 and Supplementary References [file ncomms7974-s1.pdf]

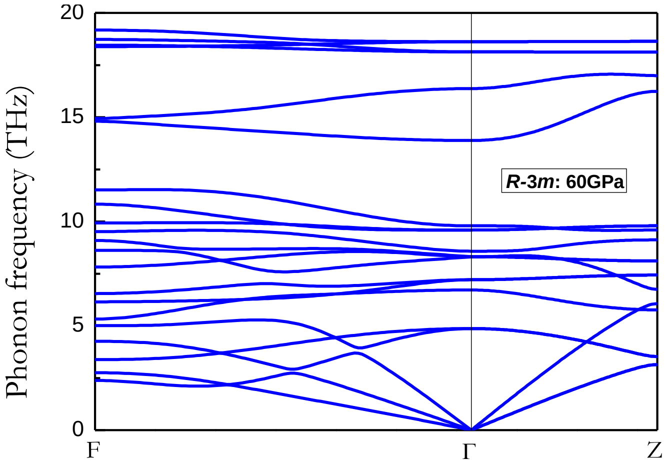

Supplementary Fig. 1. Phonon dispersion curves of *R*-3*m*-Ca_5C_2 at 60 GPa.

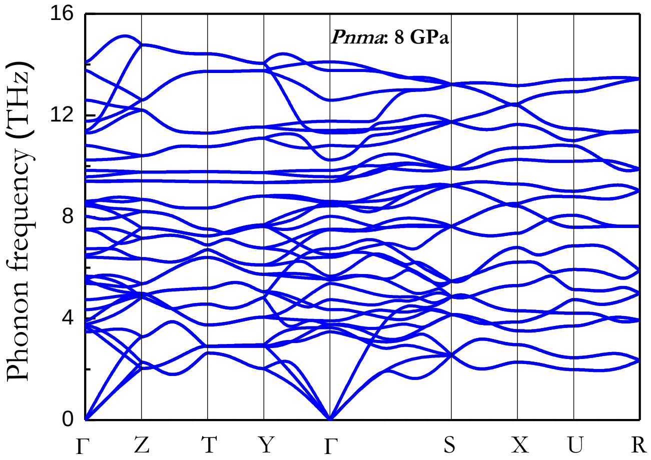

Supplementary Fig. 2. Phonon dispersion curves of *Pnma*-Ca_2C at 8GPa.

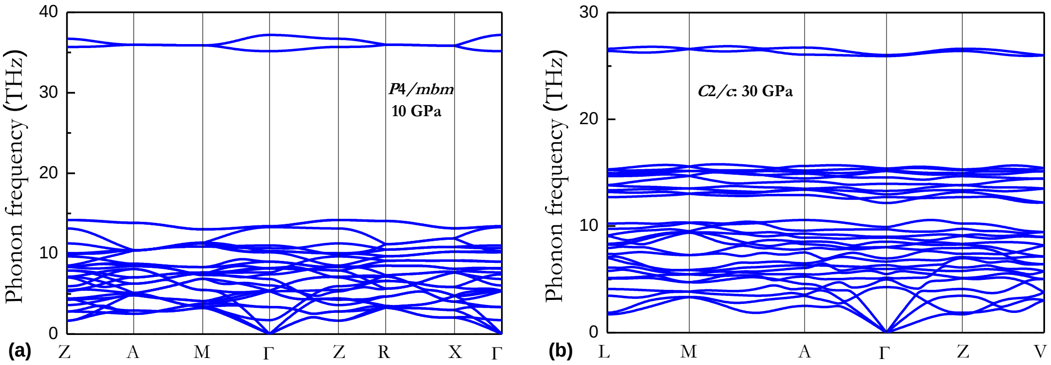

Supplementary Fig. 3. Phonon dispersion curves of Ca_3C_2. a) *P4/mbm* at 10 GPa; b) *C2/c* at 30 GPa.

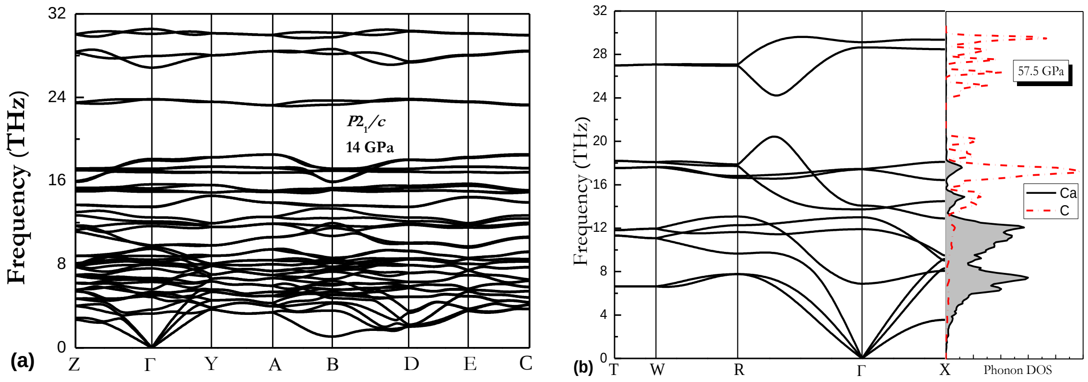

Supplementary Fig. 4. **Phonon dispersion curves of *P*2_1/*c*-CaC at 14 GPa and phonon dispersion and partial atomic phonon DOS of *Imma*-CaC at 57.5 GPa.**

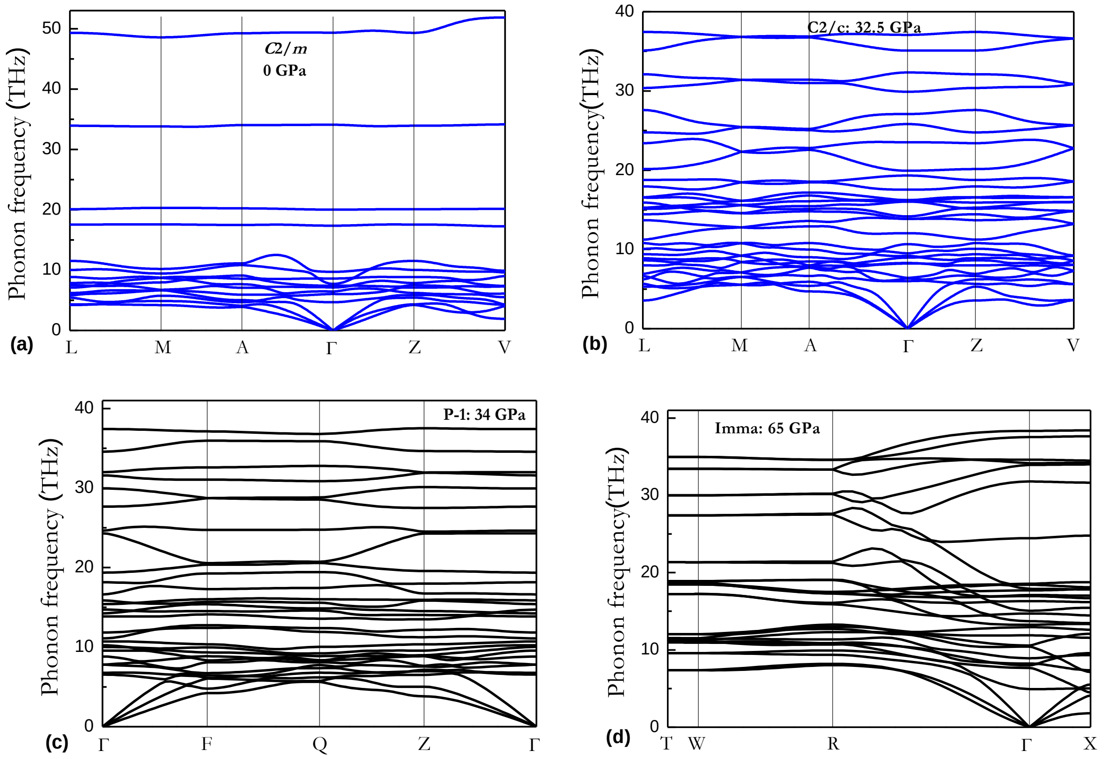

Supplementary Fig. 5 Phonon dispersion curves of *C*2/*m*-Ca_2C_3 at zero pressure (a), *C*2/*c*-Ca_2C_3 at 34.5 GPa (b), *P*-1-Ca_2C_3 at 34 GPa (c), and *Imma*-Ca_2C_3 at 65 GPa (d).

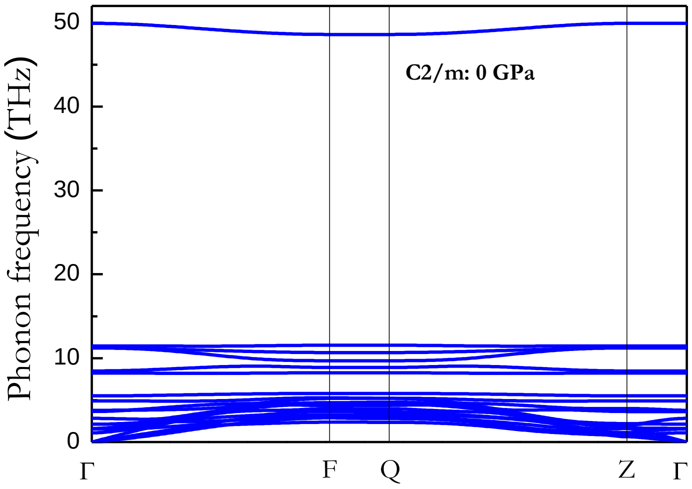

Supplementary Fig. 6. Phonon dispersion curves of *C*2/*m*-Ca_2C at zero pressure.

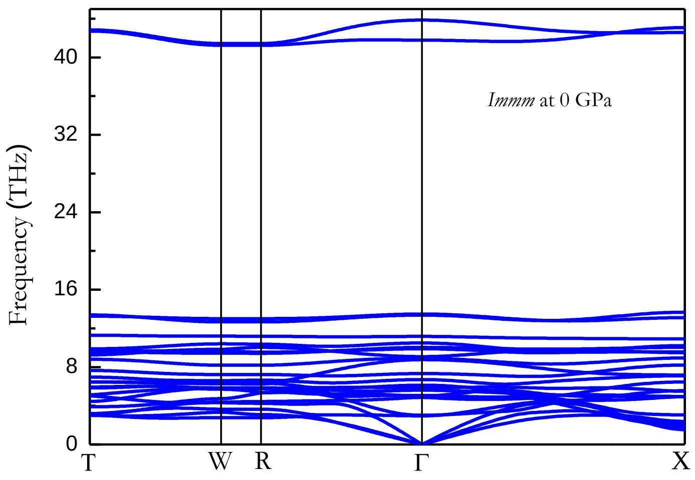

Supplementary Fig. 7. Phonon dispersion curves of CaC with *Immm* symmetry at zero pressure.

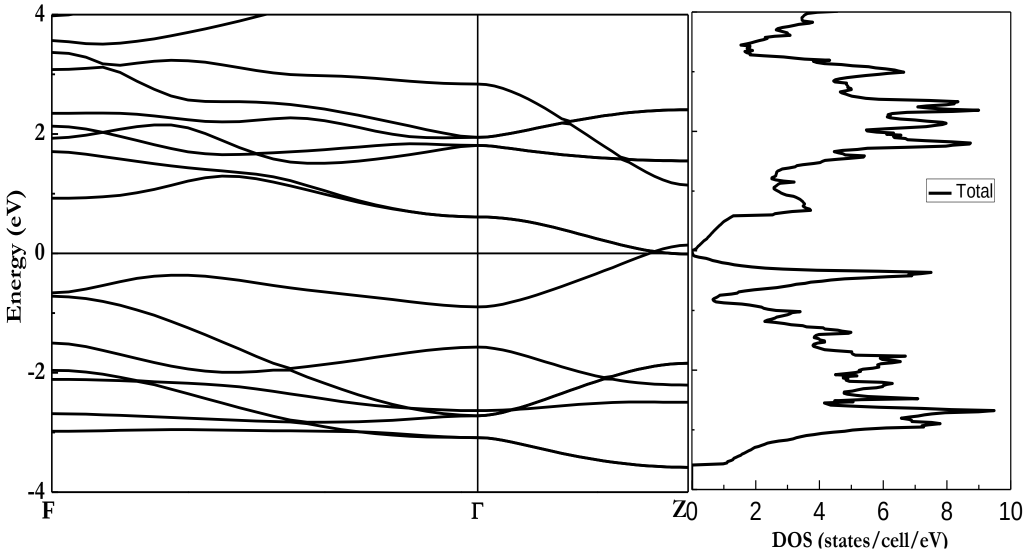

Supplementary Fig. 8. Energy band and density of states for *R*-3*m* phase of a_5C_2 solid at 60 GPa. Obviously, *R*-3*m*-C_5C_2 is a semimetal in view of a very small overlap between the bottom of the conduction band and the top of the valence band with a negligible density of states at the Fermi level.

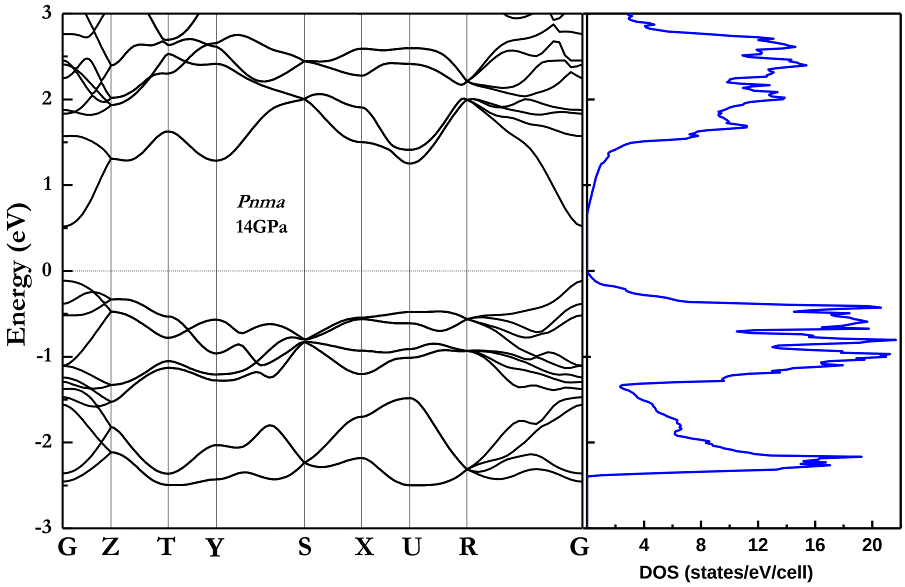

Supplementary Fig. 9. Energy band and total density of states for *Pnma* phase of Ca_2C solid at 14 GPa. One can see that it is a direct gap semiconductor.

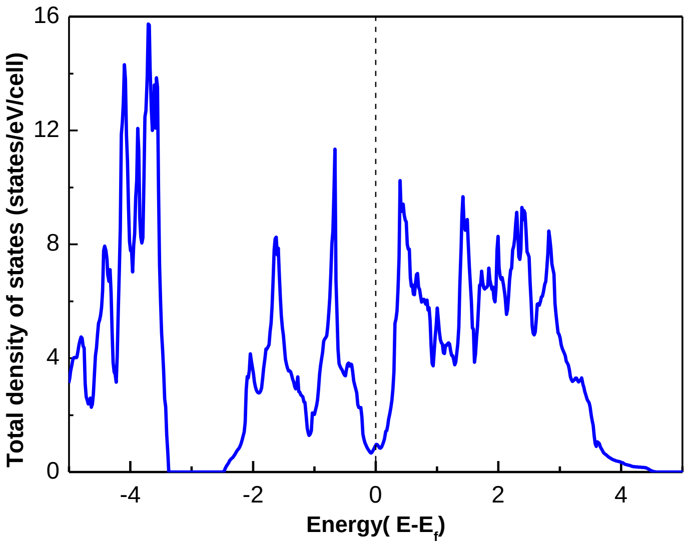

Supplementary Fig. 10. Total density of states for *C*2/*c* phase of Ca_3C_2 solid at 30 GPa. The total DOS shows that there is a deep valley close to the Fermi level and this valley is referred to as a pseudogap, indicating the presence of covalent bonding in Ca_3C_2 in agreement with the occurrence of C_2 dumbbell.

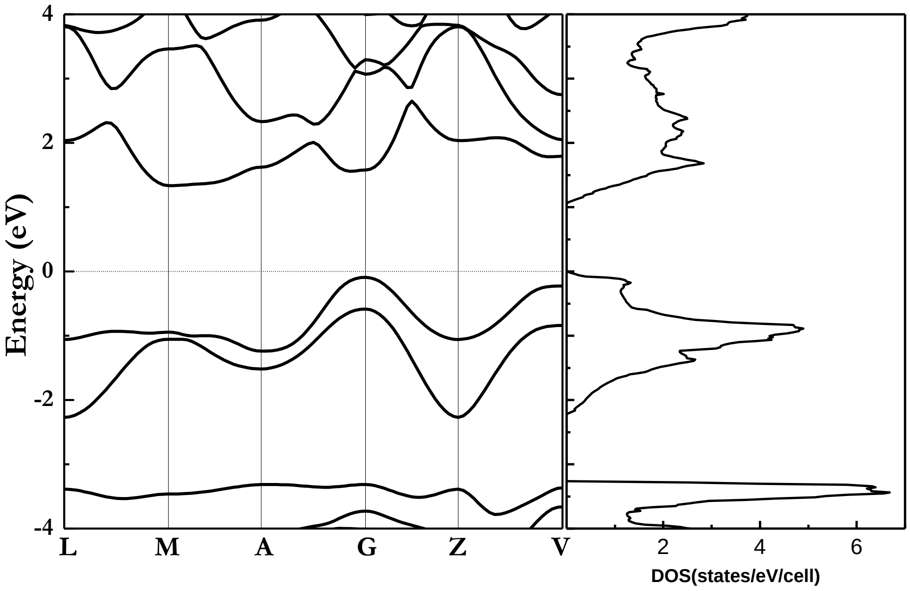

Supplementary Fig. 11. Energy band and total density of states (DOS) for *C*2/*m* phase of Ca_2C_3 at 10 GPa.

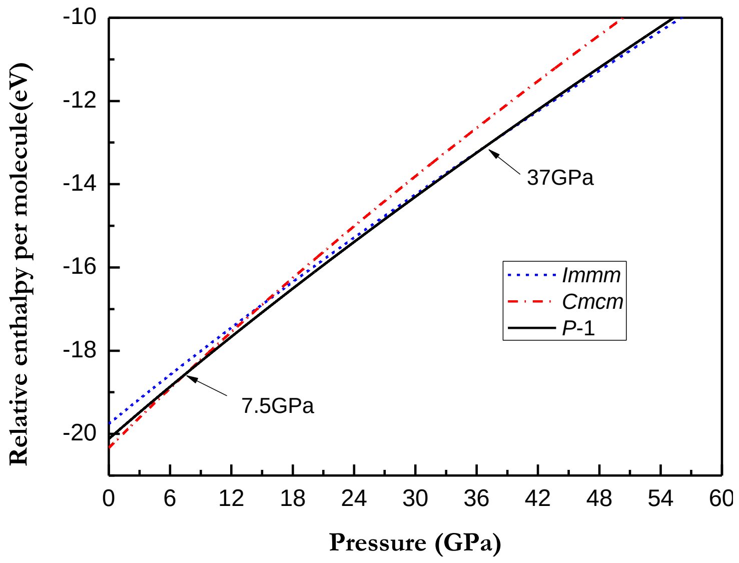

Supplementary Fig. 12. Comparison of enthalpies of different configurations of compressed CaC_2. The *C2/m* phase at ambient pressure is not given here because it transforms into *Cmcm* phase at 0.5 GPa, which has been reported in our previous work (see reference 15 in the main text).

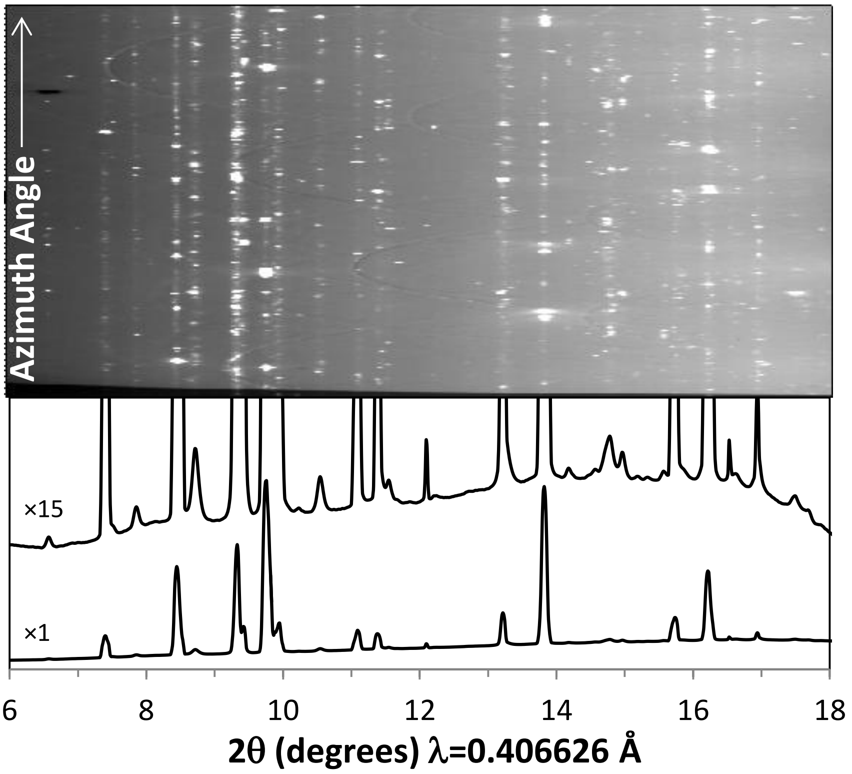

Supplementary Fig. 13. Two-dimensional diffraction image (caked) of Ca+C sample obtained at 17GPa after heating to ~2000 K (top), and corresponding integrated one-dimensional pattern (bottom).

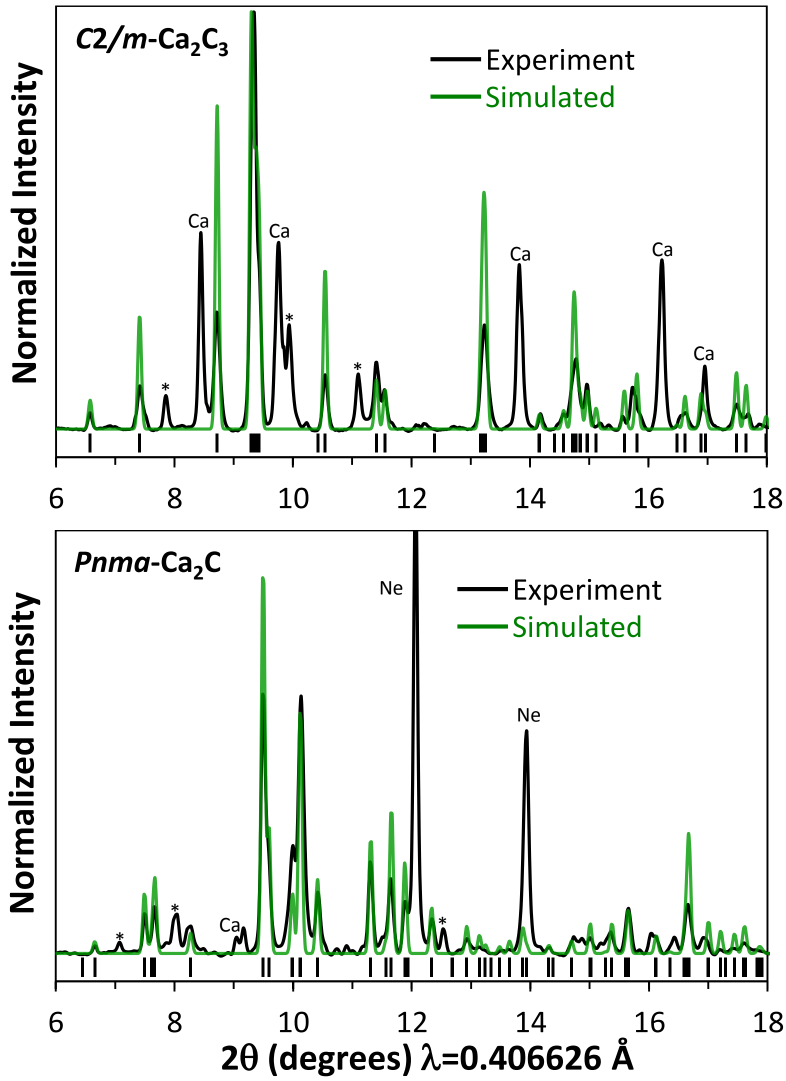

Supplementary Fig. 14. X-ray diffraction pattern obtained at 17 GPa compared with simulated intensity for the predicted *C*2/*m* structure (top) and pattern obtained at 25 GPa compared with simulated intensity for the predicted *Pnma* structure (bottom). Allowed reflection positions are indicated as vertical tick marks below the patterns. Both simulated patterns use Gaussian peak profiles and are normalized to the experimental data by the most intense peak in the structure. Reflections for Ca and Ne are labeled and unidentified peaks, which are possibly related to the tetragonal *P*4/*mbm*-Ca_3C_2 structure, are indicated by asterisks.

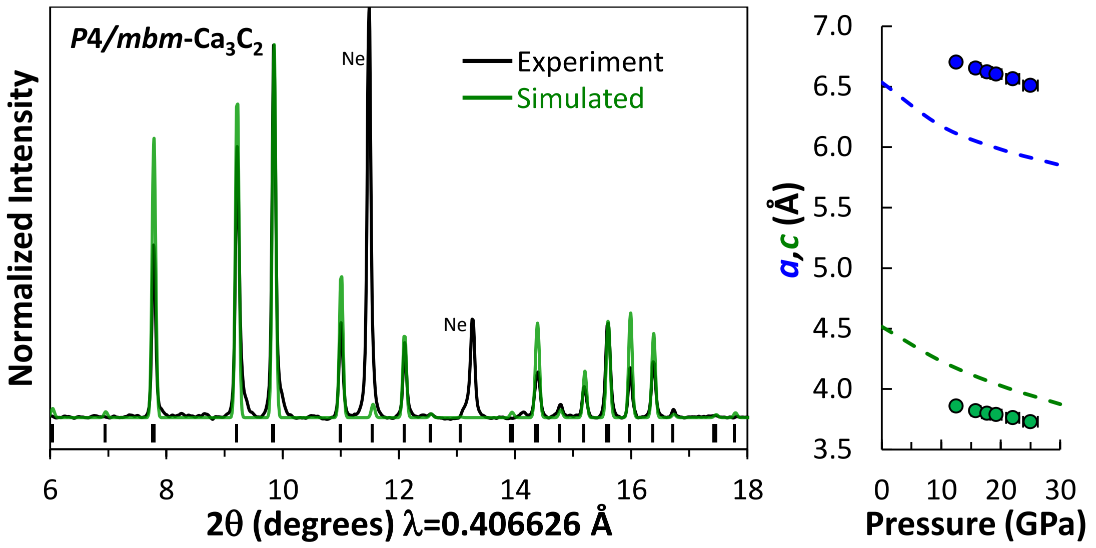

Supplementary Fig. 15. X-ray diffraction pattern obtained at 12.5 GPa compared with simulated intensities for the predicted Ca_3C_2-*P4/mbm* structure (left). Allowed reflection positions are indicated as vertical tick marks below the patterns. The simulated pattern uses Gaussian peak profiles and is normalized to the experimental data by the most intense peak in the structure. Experimental lattice parameters indexed to a tetragonal lattice as a function of pressure (points) are compared with the DFT-derived *P4/mbm*-Ca_3C_2 lattice parameters (dashed lines), showing significant deviations (right).

Supplementary Table 1. Crystal structures and Bader analyses of stable Ca-C compounds. For Ca_5C_2, Bader analysis gives charge of -0.459 for the interstitial electron density maximum at 60 GPa. For CaC_2, *P*-1 phase is a newly predicted phase with four different inequivalent carbon atoms.

Pressure (GPa)	Space group	Lattice parameters *a b c*	*α β γ*	Å °	Atomic fractional coordinates				Bader (|e|, Å^3) Charge	Volume
**Ca_5C_2**										
60	*R*-3*m*	6.8629	6.8629	6.8629	Ca1 1a	0.0000	0.0000	0.0000	1.039	10.516
	(166)	33.1725	33.1725	33.1725	Ca2 2c	0.1434	0.1434	0.1434	0.823	12.446
					Ca3 2c	0.3961	0.3961	0.3961	0.973	10.984
					C 2c	0.2642	0.2642	0.2642	-2.086	14.384
**Ca_2C**										
5	*C2/m*	6.7011	3.5874	14.6812	Ca1 4i	0.5354	0.0000	0.1297	1.237	16.570
	12	90	122.3	90	Ca2 4i	0.5158	0.0000	0.3795	-0.291	41.901
					C 4i	0.8871	0.0000	0.9686	-0.946	16.108
8	*Pnma*	6.4724	4.1903	7.5752	Ca1 4c	0.0129	0.2500	0.8341	1.163	14.257
	62	90	90	90	Ca2 4c	0.1441	0.2500	0.4102	1.158	13.344
					C 4c	0.2525	0.2500	0.0903	-2.321	23.761
**Ca_3C_2**										
20	*P4/mbm*	5.9826	5.9826	4.0329	Ca1 2a	0.0000	0.0000	0.0000	0.596	16.268
	127	90	90	90	Ca2 4h	0.1707	0.6707	0.500	0.995	13.301
					C 4g	0.4178	0.9178	0.0000	-1.293	14.651
38.7	*C2/c*	6.0129	6.4299	7.4032	Ca1 8f	0.4850	0.2126	0.0292	1.007	11.210
	15	90	123.75	90	Ca2 4e	0.0000	0.0806	0.2500	1.034	11.253
					C 8f	0.1507	0.4524	0.3195	-1.524	12.912
**CaC**										
7.1	*Immm*	12.6488	5.4600	3.7953	Ca1 2a	0.0000	0.0000	0.0000	1.288	13.418
	(71)	90	90	90	Ca2 4e	0.2600	0.0000	0.0000	1.282	13.711
					Ca3 2b	0.0000	0.5000	0.5000	0.828	21.393
					C 8n	0.3678	0.3786	0.0000	-1.170	17.206
14	*P*2$_1$/*C*	4.8201	4.7769	11.4574	Ca1 4e	0.4002	0.6034	0.1401	1.166	12.770
	14	90	128.11	90	Ca2 4e	0.2024	0.7004	0.36698	1.186	12.073
					C1 4e	0.1371	0.1091	0.0222	-0.905	12.043
					C2 4e	0.1460	0.2256	0.4153	-1.447	15.007
58	*Imma*	2.7786	3.7386	9.2294	Ca 4e	0.0000	0.2500	0.6115	1.051	12.006
	74	90	90	90	C 4e	0.0000	0.2500	0.2064	-1.051	11.963
**Ca_2C_3**										
18.1	*C2/m*	5.1511	4.9620	6.3059	Ca 4i	0.4017	0.0000	0.7208	1.346	11.891
	12	90	128.81	90	C1 4i	0.0400	0.0000	0.2283	-0.977	14.495
					C2 2a	0.0000	0.0000	0.0000	-0.738	10.025
34.5	*C2/c*	7.1370	5.5446	10.9916	Ca 8f	0.0561	0.2662	0.0961	1.188	10.927

		90	151.6	90	C1 8f	0.2797	0.1237	0.0256	-0.786	10.196
					C2 4e	0.0000	0.5071	0.2500	-0.804	9.574
40	*P*-1	4.5852	5.0227	4.6337	Ca1 2i	0.6604	0.4308	0.2407	1.135	10.601
	2	108.11	102.41	75.71	Ca2 2i	0.1836	0.0476	0.3419	1.165	10.683
					C1 2i	0.1320	0.2994	0.9755	-0.465	7.060
					C2 2i	0.1252	0.5705	0.2316	-0.940	10.213
					C3 2i	0.3776	0.0541	0.8834	-0.895	10.027
65	*Imma*	3.7753	2.6007	16.6249	Ca1 4e	0.0000	0.2500	0.2060	0.937	9.625
	65	90	90	90	Ca2 4e	0.0000	0.2500	0.9296	0.880	9.381
					C1 4e	0.0000	0.2500	0.38699	-0.494	6.557
					C2 4e	0.0000	0.2500	0.6598	-0.902	8.712
					C3 4e	0.0000	0.2500	0.4774	-0.421	6.533
					**CaC_2**					
0	*C2*/*m*	7.1712	3.8463	8.7162	Ca 4*i*	0.0425	0.0000	0.2470	1.534	13.433
		90	124.99	90	C1 4*i*	0.6063	0.0000	0.0594	-0.782	17.740
					C2 4*i*	0.5142	0.0000	0.4352	-0.752	18.067
4	*Cmcm*	3.6822	8.6324	4.7360	Ca 4*c*	0.0000	0.1465	0.2500	1.342	12.838
		90	90	90	C 8*f*	0.000	0.4376	0.1028	-0.671	12.399
15.2	*Immm*	7.0623	2.6317	6.2697	Ca 4*e*	0.2951	0.0000	0.0000	1.233	10.807
	(71)	90	90	90	C1 4*i*	0.0000	0.0000	0.2371	-0.853	10.708
					C2 4*j*	0.5000	0.0000	0.3829	-0.380	7.617
105.8	*P6/mmm*	2.5412	2.5412	3.6864	Ca 1*a*	0.0000	0.0000	0.0000	0.806	8.785
	(191)	90	90	120	C 2*d*	0.3333	0.6667	0.5000	-0.403	5.916
20	*P*-1	4.7209	4.9431	5.8359	Ca1 2i	0.1957	0.7906	0.2380	1.219	11.608
	(2)	111.93	103.03	96.56	Ca2 2i	0.1934	0.8283	0.7407	1.237	11.225
					C1 2i	0.2883	0.2749	0.1549	-0.839	11.057
					C2 2i	0.6266	0.6248	0.5625	-0.423	7.659
					C3 2i	0.4806	0.5202	0.8813	-0.368	7.528
					C4 2i	0.2756	0.2898	0.6283	-0.826	10.962

Supplementary Table 2. Carbon substructures in compressed calcium carbides, structural phase transition sequences, and electronic properties of the predicted calcium carbides. Pressure ranges of existence of each structure are given. Thermodynamically stable phases are highlighted by blue. “M” and “S” denote metal and semiconductor, respectively.

**Ca_5C_2**	isolated anion				
	**(*R*-3*m*, Semimetal)**				
	(21-100 GPa)				
**Ca_2C**	isolated dumbbells	isolated C anions			
	(*C*2/*m*, M)	**(*Pnma*, S)**			
	(0-7.5GPa)	(7.5-100GPa)			
**Ca_3C_2**	C_2 dumbells	C_2 dumbbells			
	(*P*4/*mbm*, M)	(*C*2/*c*, M, pseudogap)			
	(5-30 GPa)	(30-100 GPa)			
**CaC**	C_2 dumbbells	tetramers	chains		
	(*Immm*, M)	(*P*2$_1$/*c*, M)	(*Imma*, M)		
	(0-14 GPa)	(14-57.5GPa)	(57.5-100GPa)		
**Ca_2C_3**	C_3 trimer	chains	chains	ribbons	
	**(*C*2/*m*,S)**	(*C*2/*c*, M)	(*P*-1, M)	(*Imma*,M)	
	(0-34.5 GPa)	(34.5-40 GPa)	(40-65 GPa)	(65-100 GPa)	
**CaC_2**	C_2 dumbbells	armchair chains	stripes	ribbons	graphene
	**(*C*2/*m*,S)**	(*Cmcm*, M)	(*P*-1, M)	(*Immm*,M)	(*P*6/*mmm*,M)
	(0-0.5GPa)	(0.5-7.5 GPa)	(7.5-37GPa)	(37-105.8GPa)	(>105.8GPa)

Supplementary Table 3. Experimental and density functional theory (DFT) lattice parameters for monoclinic *C*2/*m* structure of Ca_2C_3 with pressure.

P (GPa)	*a* (Å)	*b* (Å)	*c* (Å)	β (deg)	V (Å^3)
Experiment					
17.1(9)	5.169(4)	4.994(3)	6.322(3)	128.53(3)	127.64(4)
15.4(8)	5.185(9)	5.015(7)	6.340(7)	125.56(7)	128.9(1)
14.1(7)	5.189(5)	5.041(4)	6.341(5)	128.46(4)	129.9(1)
10.3(5)	5.225(6)	5.090(5)	6.382(6)	128.36(6)	133.1(1)
8.2(4)	5.246(4)	5.114(3)	6.419(4)	128.32(3)	135.13(8)
6.9(3)	5.260(8)	5.154(3)	6.422(7)	128.27(6)	136.7(2)
5.1(3)	5.265(6)	5.223(6)	6.447(6)	128.25(5)	139.3(1)
0.10(5)	5.392(5)	5.298(4)	6.574(9)	128.19(6)	147.6(2)
DFT					
0	5.3992	5.3095	6.5742	128.34	147.81
10	5.2408	5.0838	6.4037	128.56	133.42
18.1	5.1511	4.9620	6.3059	128.81	125.59
20	5.1321	4.9362	6.2852	128.87	123.97
30	5.0472	4.8261	6.1953	129.22	116.91

Supplementary Table 4. Experimental and DFT lattice parameters for orthorhombic *Pnma* structure of Ca_2C with pressure.

P (GPa)	a (Å)	b (Å)	c (Å)	V (Å^3)
**Experiment**				
24(1)	6.122(1)	4.004(1)	7.223(1)	177.04(3)
22(1)	6.168(6)	4.032(3)	7.266(6)	180.7(2)
15.0(8)	6.280(6)	4.081(3)	7.396(6)	189.6(2)
10.1(5)	6.449(5)	4.157(4)	7.523(7)	201.7(2)
5.1(3)	6.502(3)	4.265(2)	7.632(4)	211.6(1)
**DFT**				
0	6.6886	4.3893	7.9813	234.32
10	6.4146	4.1542	7.5186	200.35
20	6.1582	4.0211	7.3250	181.39
30	5.9296	3.9337	7.2045	168.04

Supplementary Note 1

It is well known that the stability of a solid phase of solid at zero temperature depends on both low enthalpy and dynamic stability. The latter is justified by phonon spectra which were calculated by using first-principles total energy calculations together with Phonopy code. The calculated phonon spectra of stable structures of newly predicted compounds are presented in Supplementary Figs. 1-7. The absence of imaginary frequencies observed in phonon spectra indicates their dynamical stability.

Supplementary Note 2

Two-dimensional X-ray diffraction patterns were obtained using a MAR345 image plate, with distance, center, rotation and tilt calibrated using a high-purity CeO_2 standard, as implemented within FIT2D[2]. The observed intensities of the Debye-Scherrer diffraction rings obtained from laser-heated Ca-C samples showed significant azimuthal intensity variation (*i.e.*, “spotty” rings), indicating poor powder averaging statistics and a distribution of grain sizes comparable to the beam diameter (Supplementary Fig. 13).

A number of masking options were performed within FIT2D, including the implementation of a threshold mask and peak/polygon masks, to remove regions of saturated intensity (65,000 counts). While this procedure yielded integrated one-dimensional patterns with reasonable intensities (we compared experimental intensities with intensities derived from *ab initio* atomic positions), this intensity information was not suitable for Rietveld structural refinements as it was not representative of an ideal powder, which assumes a perfectly random distribution of crystallites in all orientations. Therefore, we used the Le Bail intensity extraction method with full profile refinements, as implemented in GSAS with EXPGUI[3-4], to obtain lattice parameters and to compare data with *ab initio* structure predictions.

Despite the lack of ideal powder XRD data suitable for precise Rietveld refinements

of atomic positions, intensities derived from integrated one-dimensional diffraction patterns were comparable with simulated diffraction intensities based on *ab initio* structural models. Supplementary Fig. 14 compares the integrated diffraction intensities from Supplementary Fig. 13, and another pattern obtained at 25 GPa, with the intensities simulated from the DFT structural models for *C*2/*m*-Ca_2C_3 and *Pnma*-Ca_2C (represented by Gaussian peak profiles), normalized to the most intense peak from each phase. One can observe good semi-quantitative agreement between the experimental and simulated diffraction intensities for all allowed reflections in these structures, indicating that the actual experimental diffraction intensities are consistent with the atomic positions from the DFT structural models. These observations, combined with the excellent quantitative agreement between experimental and calculated lattice parameters, confirm the formation of these phases.

In addition to the formation of *C*2/*m*-Ca_2C_3 and *Pnma*-Ca_2C, based on semi-quantitative intensity agreement of all allowed reflections and excellent quantitative agreement between experimental and calculated lattice parameters, Bragg reflections from a third phase were identified in some diffraction patterns (indicated by asterisks in Supplementary Fig. 14). In some samples we were able to isolate diffraction from this phase by translating the laser-heated sample position several microns, with respect to the synchrotron X-ray beam. Supplementary Fig. 15 shows an example of this third Ca-C phase obtained at 12.5 GPa after heating at ~2000 K. This phase could be indexed to a tetragonal lattice with a =6.703(3) and c = 3.858(4) Å and was compared with DFT structural predictions. The intensities of individual Bragg reflections were in very good agreement with simulated intensities for the *P*4/*mbm*–Ca_3C_2 structure (Supplementary Fig. 15), however, the lattice parameters were significantly different, *e.g.*, at 20 GPa calculated values for a and c differ from experiment by approximately -9% and +6%, respectively (For Ca_2C_3 and Ca_2C agreement between experimental and calculated lattice parameters was always less than 1%). Therefore, this structure is likely related to the tetragonal Ca_3C_2 structure type (or the calcium sublattice within) based on intensity agreement, but the actual structure and composition cannot be unambiguously confirmed at present and will be

the topic of subsequent studies.

Lattice parameters for *C*2/*m*-Ca_2C_3 and *Pnma*-Ca_2C were determined though full profile refinement using the Le Bail intensity extraction method, as implemented in GSAS with EXPGUI[3,4]. Background profiles were initially estimated graphically, and then refined as shifted Chebyschev polynomials (GSAS function #1). Three pseudo-Voigt profile parameters (GU, GW and LX) were refined iteratively, in addition to the unit cell refinement variables. In patterns where significant diffraction intensity was observed from multiple peaks of the tetragonal *P*4/*mbm*-Ca_3C_2-like structure, this phase was included in the refinements, otherwise this phase was neglected. Supplementary Tables 3 and 4 summarize the experimentally refined lattice parameters for *C*2/*m*-Ca_2C_3 and *Pnma*-Ca_2C, as well as lattice parameters obtained from DFT calculations. Experimental pressures were determined from the equation of state of Neon[5] above its solidification pressure, or from ruby fluorescence[6]. The maximal uncertainty in pressure is reported as the largest of either 5% of the absolute pressure or the difference between the pressures measured from Ruby and Ne.

Supplementary References

1. Klimeš, J., Bowler, D. R. & Michaelides, A. Chemical accuracy for the van der Waals density functional. *J. Phys. Cond. Matt.* **22**, 022201 (2010).
2. Hammersley, A. P. FIT2D: An Introduction and Overview. *ESRF Internal Report* ESRF97HA02T (1997).
3. Larson, A. C. & Von Dreele, R. B. Los Alamos National Laboratory Report LAUR. 86-748 (1994).
4. Toby, B. H. EXPGUI, a graphical user interface for GSAS. *J. Appl. Crystallogr.* **34**, 210-213 (2001).
5. Hemley, R. J. *et al.* X-ray diffraction and equation of state of solid neon to 110 GPa. *Phys. Rev. B* **39**, 11820-11827 (1989).
6. Mao, H. K., Xu, J. & Bell, P. M. Calibration of the ruby pressure gauge to 800 kbar under quasi-hydrostatic conditions. *J. Geophy. Res.: Solid Earth* **91**, 4673-4676 (1986).
